# The immunologic advantage of recurrent nasopharyngeal carcinoma from the viewpoint of Galectin-9/Tim-3-related changes in the tumour microenvironment

**DOI:** 10.1038/s41598-017-10386-y

**Published:** 2017-09-04

**Authors:** Tseng-Cheng Chen, Chao-Hsien Chen, Cheng-Ping Wang, Pei-Hsuan Lin, Tsung-Lin Yang, Pei-Jen Lou, Jenq-Yuh Ko, Chen-Tu Wu, Yih-Leong Chang

**Affiliations:** 1 Department of Otolaryngology, National Taiwan University Hospital and National Taiwan University College of Medicine, Taipei, 10002 Taiwan; 2 Department of Immunology, The University of Texas MD Anderson Cancer Center, Houston, 77030 Texas United States; 3 The University of Texas Graduate School of Biomedical Sciences at Houston, Houston, 77030 Texas United States; 4 Department of Otolaryngology, National Taiwan University Hospital Yun-Lin Branch, YunLin County, 640 Taiwan; 5 Graduate Institute of Pathology, National Taiwan University College of Medicine, Taipei, 10002 Taiwan; 6 Department of Pathology, National Taiwan University Hospital and National Taiwan University College of Medicine, Taipei, 10002 Taiwan

## Abstract

Given salvage treatment for recurrent nasopharyngeal carcinoma (NPC) remains a clinical dilemma, immunotherapy targeting NPC-specific immunosuppression may bring new hope. We analyzed the expression of CD8, CD4, Foxp3 and Tim-3 in lymphocytes, and of Galectin-9 in tumour cells between paired primary and recurrent NPC from 95 patients and we noted that there was significant increase in the expression of Galectin-9+ tumour cells (p < 0.001) and Foxp3+ lymphocytes (p < 0.001) but a significant decrease in the expression of CD8+ lymphocytes (p = 0.01) between paired primary and recurrent NPC. Of all patients, 53 patients (55.79%) and 57 patients (60%) had increased percentages of Galectin-9+ tumour cells and of Foxp3+ lymphocytes, respectively. Conversely, 42 patients (44.21%) had decreased percentages of CD8+ lymphocytes. The patients with high Galectin-9 expression in recurrent NPC frequently also had high Tim-3 (p = 0.04) and Foxp3 (p = 0.01), and low CD8 (p = 0.04) expression in lymphocytes. After multivariate analyses, low CD8 expression in lymphocytes was an independent risk factor for relapse-free survival (p = 0.002) and overall survival (p = 0.02). Our data suggests that recurrent NPC may had more immunologic advantage than primary NPC, especially the Galectin-9/Tim-3 pathway. The immunotherapies targeting Galectin-9/Tim-3/Foxp3 interaction may serve as a potential salvage treatment for recurrent NPC.

## Introduction

In contrast to other head and neck squamous cell carcinomas, nasopharyngeal carcinoma (NPC) is characterized by its close association with Epstein-Barr virus (EBV) infection, poor differentiation of tumour cells and high susceptibility to radiotherapy and chemotherapy^1, 2^. Due to its high sensitivity to radiation, the mainstream treatment for primary NPC is always radiotherapy with or without chemotherapy^3^. However, some patients may experience recurrent disease and must undergo salvage treatment^4–6^. In general, recurrent NPC responds to treatment differently than primary NPC. First, it is easy to speculate that recurrent NPC might become more radioresistant than primary NPC^7^. Second, the current choices of salvage treatment such as reirradiation, salvage nasopharyngectomy or other multiple modalities frequently result in severe delayed toxicities, such as cranial nerve damage, radionecrosis or temporal lobe necrosis, which can seriously affect quality of life in patients with recurrent NPC^8^. Most importantly, there has not been a specific consensus regarding the salvage treatment for recurrent NPC. Therefore, recurrent NPC still represents a clinical dilemma and a challenging problem for clinicians.

In recent years, the progress in immunotherapy has opened new fields in cancer treatment. For example, multiple immunosuppressive mechanisms in NPC have been proposed, including interleukin-10, transforming growth factor β1, Fas ligand, Foxp3+ regulatory T cells and Galectin-9^9**–**14^. The Galectin-9/Tim-3 axis had been reported to be one of the NPC-specific immunosuppression pathway^13^. The immunotherapeutic drug targeting this axis are also available now^15^. Therefore, it is interesting and important to discover the changes of Galectin-9/Tim-3 pathway and generalized immunologic status (CD8+ , CD4+ and Foxp3+ lymphocytes in tumour microenvironment) between primary and recurrent NPC. The change in immunologic status between primary and recurrent NPC may have clinical implication in understanding the possibility of immunotherapy for the recurrent NPC patients. Therefore, we evaluated the paired immunologic expression of CD8+ lymphocytes, CD4+ lymphocytes, Foxp3+ lymphocytes, and the Galectin-9/Tim-3 pathway in the tumour microenvironment between primary and recurrent NPC from 95 patients.

Immunotherapy targeting NPC-specific immunosuppression may bring new hope for patients with recurrent NPC. For clinicians, the question of whether immunotherapy could be the choice of salvage treatment is imperative to answer. To answer this question, we analyzed the immunologic changes in the tumour microenvironment between primary and recurrent NPC. The scientific aim of our study is to evaluate the consequential changes in tumour immunology from chemotherapy and radiotherapy of primary NPC and understand the possible clinical implication of immunotherapeutic potential for patients with recurrent NPC in the future.

## Results

### Patient demographics

A total of 95 eligible patients with recurrent NPC were enrolled in this study, including 70 male and 25 female patients. Their ages ranged from 27 to 77 years, with a mean age of 50 ± 11 years at diagnosis. The length of time between when primary treatment for NPC ended and diagnosis of recurrent NPC occurred ranged from 1 to 163 months, with a mean of 26 ± 26 months. The overall follow-up period was from 12 to 174 months, with a mean of 59 ± 27 months. The recurrent NPC profile of the 97 patients are as follows: 43 patients had local recurrence in nasopharynx, 12 patients had local and regional neck recurrence, 11 patients had regional neck recurrence, 11 patients had distant Lung metastases, 5 patients had distant Bone metastases, 5 patients had multiple (≥2) distant metastases, 3 patients had distant Liver metastases, 3 patients had distant Spinal metastases and 2 patients had recurrence over parotid gland. The basic characteristics, clinical characteristics and changes of immunologic markers between primary and recurrent NPC are listed in Table 1.

**Table 1:** Clinical characteristics, Histopathological characteristics and Immunologic expression in primary NPC and recurrent NPC in our series.

Characteristics	Primary NPC (n = 95)	Recurrent NPC (n = 95)	p value
Age (years)	50 ± 11		
Gender	70 males, 25 females		
Histological types			
Keratinizing squamous cell carcinoma	4/95 (4.21%)		
Non-keratinizing carcinoma, differentiated	20/95 (21.05%)		
Non-keratinizing carcinoma, undifferentiated	71/95 (74.74%)		
Tumour stages			
Early stages (I/II/III)	45/95 (47.37%)	52/95 (54.74%)	0.38
Advanced stages (IVA/IVB)	50/95 (52.63%)	43/95 (45.26%)	
Changes of Galectin-9+ tumour cells			
Increased	53/95 (55.79%)		
Same	22/95 (23.16%)		
Decreased	20/95 (21.05%)		
Changes of CD8+ lymphocytes in TM			
Increased	24/95 (25.26%)		
Same	29/95 (30.53%)		
Decreased	42/95 (44.21%)		
Changes in Foxp3+ lymphocytes in TM			
Increased	57/95 (60%)		
Same	15/95 (15.79%)		
Decreased	23/95 (24.21%)		
Changes of CD4+ lymphocytes in TM			
Increased	38/95 (40%)		
Same	24/95 (25.26%)		
Decreased	33/95 (34.74%)		
Changes of Tim-3+ lymphocytes in TM			
Increased	34/95 (35.79%)		
Same	25/95 (26.32%)		
Decreased	36/95 (37.89%)		

Abbreviation: NPC, nasopharyngeal carcinoma; TM: tumour microenvironment.

### Changes in immunologic marker expression from primary to recurrent NPC

**Figure 1:**
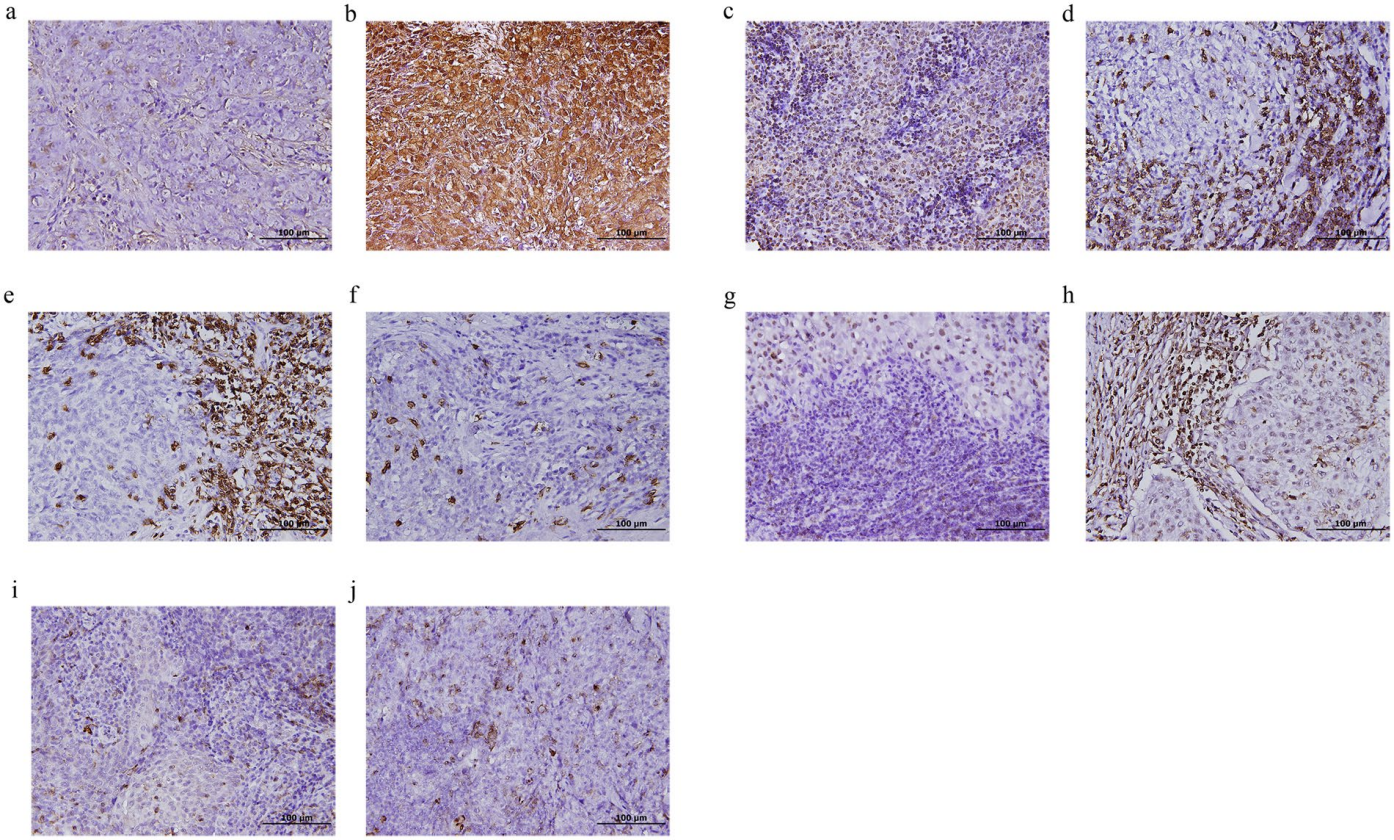
The immunohistochemical staining of different immunologic markers in paired initial and recurrent nasopharyngeal carcinoma (NPC) tissue. (**a**) Initial non-keratinizing NPC, undifferentiated with low level of Galectin-9 expression in tumour cells (0%). (**b**) The liver metastatic carcinoma, undifferentiated with high level of Galectin-9 expression in tumour cells (80%). (**c**) Initial non-keratinizing NPC, undifferentiated with low level of Foxp3 expression in stromal infiltrating lymphocytes (5%). (**d**) The contralateral neck nodal recurrent carcinoma with high level of Foxp3 expression in stromal infiltrating lymphocytes (70%). (**e**) Initial non-keratinizing NPC, differentiated with high level of CD8 expression in stromal infiltrating lymphocytes (80%). (**f**) The ipsilateral neck nodal recurrent carcinoma with low level of CD8 expression in stromal infiltrating lymphocytes (15%). (**g**) Initial non-keratinizing NPC, undifferentiated with low level of CD4 expression in stromal infiltrating lymphocytes (10%). (**h**) The lung metastatic carcinoma, undifferentiated with high level of CD4 expression in stromal infiltrating lymphocytes (80%). (**i**) Initial non-keratinizing NPC, undifferentiated with modest Tim-3 expression in stromal infiltrating lymphocytes (20%). (**j**) The lung metastatic carcinoma, undifferentiated with mildly increased Tim-3 expression in stromal infiltrating lymphocytes (30%).

When we compared the expression of all immunologic markers (Fig. 1) in the tumour microenvironment between primary and recurrent NPC, we found significant differences in the mean percentages of Galectin-9+ tumour cells, Foxp3+ lymphocytes and CD8+ lymphocytes. The means and standard deviations of the percentages of Galectin-9+ tumour cells in primary and recurrent NPC were 29.41 ± 27.87% and 41.14 ± 29.60%, respectively (p = 0.006). The means and standard deviations of the percentages of Foxp3+ lymphocytes in primary and recurrent NPC were 25.65 ± 21.70% and 37.54 ± 28.23%, respectively (p = 0.01). The means and standard deviations of the percentages of CD8+ lymphocytes in primary and recurrent NPC were 17.85 ± 18.99% and 11.61 ± 15.11%, respectively (p = 0.02). On the contrary, there were no significant difference in the mean percentages of CD4+ lymphocytes and Tim-3+ lymphocytes. The means and standard deviations of the percentages of CD4+ lymphocytes in primary and recurrent NPC were 14.91 ± 19.09% and 17.12 ± 21.27%, respectively (p = .74). The means and standard deviations of the percentages of Tim-3+ lymphocytes in primary and recurrent NPC were 15.88 ± 15.52% and 14.38 ± 14.50%, respectively (p = .43).

**Figure 2:**
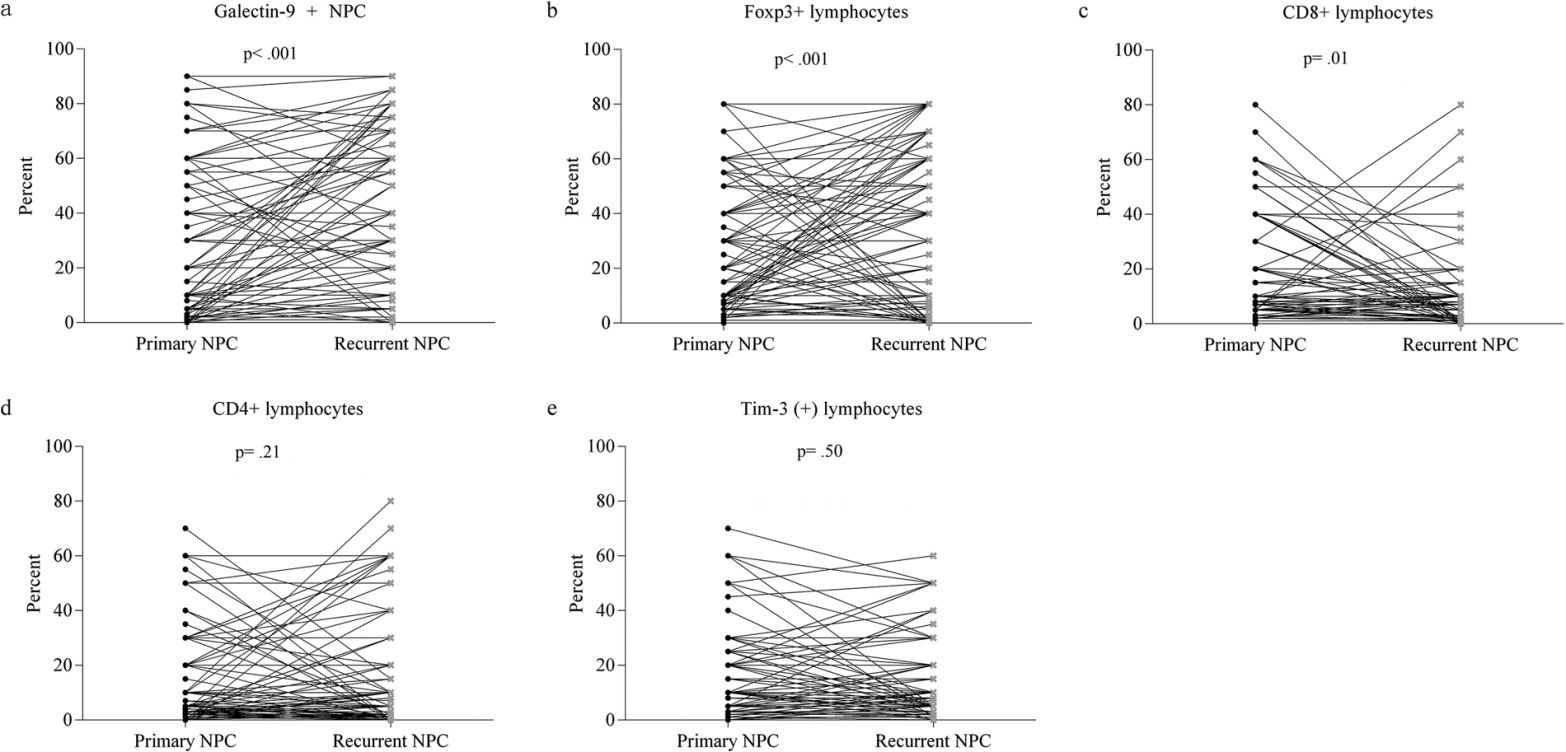
The changes in the percentages of (**a**) Galectin-9+ tumour cells (significantly increased), (**b**) Foxp3+ lymphocytes (significantly increased), (**c**) CD8+ lymphocytes (significantly decreased), (**d**) CD4+ lymphocytes (increased) and (**e**) Tim-3+ lymphocytes (decreased) in the paired primary and recurrent nasopharyngeal carcinoma tissues.

Furthermore, the differences in the expression of these immunologic markers between primary and recurrent NPC were verified by Wilcoxon signed-rank test. All changes in the expression of the tested immunologic markers in the paired samples are shown in Fig. 2. Again, there were significant differences in the percentages of Galectin-9+ tumour cells, Foxp3+ lymphocytes and CD8+ lymphocytes between the paired primary and recurrent NPC. The percentages of Galectin-9+ tumour cells and Foxp3+ lymphocytes increased significantly (p < 0.001 and p < 0.001, respectively) in recurrent NPC. Conversely, the percentage of CD8+ lymphocytes decreased significantly in recurrent NPC (p = 0.01).

### The different patterns of immunologic markers between patients with low and high Galectin-9 expression levels in recurrent NPC

The basic characteristics and clinicopathological data of the patients with high and low Galectin-9 expression levels in recurrent NPC are shown in Table 2. There were no other significant differences between the groups with low and high Galectin-9 expression. However, the expression patterns of specific immunologic markers including CD8, Tim-3 and Foxp3 in lymphocytes differed in the group with high Galectin-9 expression (Table 3) from those with low Galectin-9 expression. In summary, the patients with high Galectin-9 expression in recurrent NPC was associated with a higher percentage of Tim-3+ lymphocytes and a more immunocompromised status (a lower percentage of CD8+ and higher percentage of Foxp3+ lymphocytes) in the tumour microenvironment.

**Table 2:** The characteristics and clinicopathological data of all patients with nasopharyngeal carcinoma in our series.

	Low Galectin-9 in recurrent NPC (n = 45)	High Galectin-9 in recurrent NPC (n = 50)	p value
Age (years)			
>50	23/45 (51.11%)	22/50 (44%)	0.54
≤50	22/45 (48.89%)	28/50 (56%)	
Gender			
Female	8/45 (17.78%)	17/50 (34%)	0.10
Male	37/45 (82.22%)	33/50 (66%)	
Histological types			
Keratinizing squamous cell carcinoma	2/45 (4.44%)	2/50 (4%)	0.25*
Non-keratinizing carcinoma, differentiated	6/45 (13.33%)	14/50 (28%)	
Non-keratinizing carcinoma, undifferentiated	37/45 (82.22%)	34/50 (68%)	
Type of recurrence			
Primary sites (local nasopharynx)	22/45 (48.89%)	21/50 (42%)	0.92
Regional sites (Neck lymph node)	5/45 (11.11%)	6/50 (12%)	
Distant metastases	13/45 (28.89%)	16/50 (32%)	
Both Primary and Regional sites	5/45 (11.11%)	7/50 (14%)	
Initial stages			
Early stages (I/II/III)	24/45 (53.33%)	21/50 (42%)	0.31
Advanced stages (IVA/IVB)	21/45 (46.67%)	29/50 (58%)	
Recurrent stages			
Early stages (rI/rII/rIII)	27/45 (60%)	27/50 (54%)	0.41
Advanced stages (rIVA/rIVB/rIVC)	18/45 (40%)	23/50 (46%)	
Initial treatment modalities			
CCRT	32/45 (71.11%)	28/50 (56%)	0.14
Induction C/T + CCRT	13/45 (28.89%)	22/50 (44%)	
Salvage treatment modalities			
Surgery only	14/45 (31.11%)	17/50 (34%)	0.08*
Re-CCRT	13/45 (28.89%)	5/50 (10%)	
Salvage C/T only	14/45 (31.11%)	25/50 (50%)	
Multiple modalities	4/45 (8.89%)	3/50 (6%)	

Abbreviation: CCRT, concurrent chemoradiotherapy; C/T: chemotherapy; NPC, nasopharyngeal carcinoma; *using Fisher’s exact test.

**Table 3:** The distribution and association of different immunologic factors in recurrent nasopharyngeal carcinoma and tumour-infiltrating lymphocytes.

	Low Galectin-9 in recurrent NPC (n = 45)	High Galectin-9 in recurrent NPC (n = 50)	Odds ratio (95% CI)	p value
Expression of CD8+ lymphocytes				
Low	19/45 (42.22%)	32/50 (64%)	2.43 (1.06~5.56)	0.04
High	26/45 (57.78%)	18/50 (36%)		
Expression of Foxp3+ lymphocytes				
Low	27/45 (60%)	17/50 (34%)	2.91 (1.26~6.71)	0.01
High	18/45 (40%)	33/50 (66%)		
Expression of Tim-3+ lymphocytes				
Low	28/45 (62.22%)	20/50 (40%)	2.47 (1.08~5.65)	0.04
High	17/45 (37.78%)	30/50 (60%)		
Expression of CD4+ lymphocytes				
Low	24/45 (53.33%)	24/50 (48%)	1.24 (0.55~2.77)	0.68
High	21/45 (46.67%)	26/50 (52%)		

Abbreviation: NPC, nasopharyngeal carcinoma; CI, confidence interval.

### Immunologic expression had a significant association with relapse-free survival and overall survival

**Figure 3:**
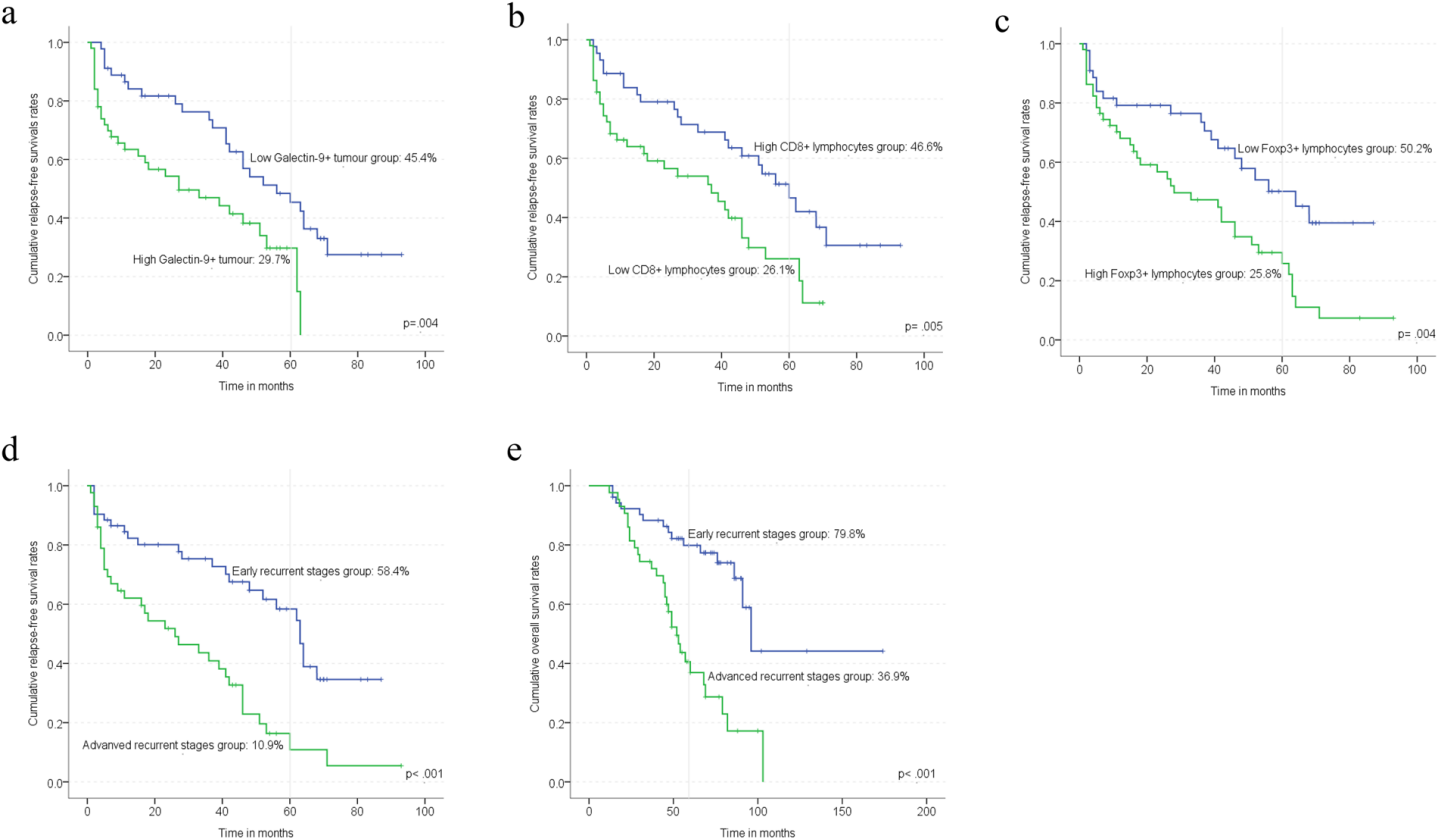
The relapse-free survival and overall survival curves with significant difference (p < 0.05). The median percentages of all immunologic markers were used as the cut-off points for grouping high or low level of expression groups. (**a**) Recurrent patients with low or high Galectin-9+ tumour, relapse-free survival curves (**b**) Recurrent patients with low or high CD8+ lymphocytes, relapse-free survival curves. (**c**) recurrent patients with low or high Foxp3+ lymphocytes, relapse-free survival curves. (**d**) Patients with early or advanced recurrent stages, relapse-free survival curves. (**e**) patients with early or advanced recurrent stages, overall survival curves

In the univariate analysis (Table 4), a high percentage of Galectin-9+ tumour cells (p = 0.004), a low percentage of CD8+ lymphocytes (p = 0.005) and a high percentage of Foxp3+ lymphocytes (p = 0.004) in the recurrent tumour microenvironment as well as in the advanced stages of recurrent NPC (p < 0.001) were identified as significant risk factors for relapse-free survival. For overall survival, only an advanced stage of recurrent NPC (p < 0.001) was a significant risk factor. The relapse-free survival and overall survival curves with significant difference (p < 0.05) are shown in Fig. 3.

**Table 4:** Univariate analysis of possible risk factors for survival.

Characteristics	Relapse-free survival		Overall survivals	
	Rates	p values	Rates	p value
Ages (years)				
>50	41.7%	0.97	58.8%	0.36
≤50	31.9%		62.8%	
Gender				
Male	40.4%	0.21	60.2%	0.43
Female	22.5%		62.4%	
Histological types				
Nonkeratinizing carcinoma, undifferentiated	29.5%	0.26	75%	0.48
Nonkeratinizing carcinoma, differentiated	59.7%		66.9%	
Keratinizing squamous cell carcinoma	75%		57.9%	
Recurrent NPC Galectin-9 expression				
Low	45.4%	0.004	69.4%	0.06
High	29.7%		53.2%	
TILs CD8+ lymphocytes				
Low	26.1%	0.005	53.5%	0.06
High	46.6%		69.1%	
TILs CD4+ lymphocytes				
Low	44.6%	0.21	65.1%	0.20
			56.5%	
High	29.5%			
TILs Foxp3+ lymphocytes				
Low	50.2%	0.004	68.9%	0.08
			53.8%	
High	25.8%			
TILs Tim-3+ lymphocytes				
Low	42.2%	0.32	61.8%	0.65
High	31.0%		59.7%	
Initial stage				
Early stages (I/II/III)	35%	0.33	58.4%	0.36
			63.1%	
Advanced stages (IVA/IVB)	39.1%			
Recurrent stage				
Early stages (rI/rII/rIII)	58.4%	<0.001	79.8%	<0.001
Advanced stages (rIVA/rIVB/rIVC)	10.9%		36.9%	

Abbreviation: NPC, nasopharyngeal carcinoma; TILs, tumour-infiltrating lymphocytes.

All significant risk factors for both relapse-free survival and overall survival were further examined by multivariate analysis (Table 5). This analysis revealed that an advanced stage of recurrent disease (hazard ratio (HR): 2.58, p = 0.001) and a low percentage of CD8+ lymphocytes (HR: 2.40, p = 0.002) were independent risk factors for worse relapse-free survival. For overall survival, the analysis also revealed that an advanced stage of recurrent disease (HR: 3.92, p < 0.001) and a low percentage of CD8+ lymphocytes (HR: 2.16, p = 0.02) were independent risk factors. In short, the patients with recurrent NPC who had an advanced stage of disease or a low percentage of CD8+ lymphocytes in the tumour microenvironment had significantly worse relapse-free survival and overall survival.

**Table 5:** Multivariate analysis of possible risk factors using the Cox logistic regression method.

Characteristics	Relapse-free survival		Overall survival	
	HR (95% CI)	P value	HR (95% CI)	p value
Recurrent Stages				
Advanced stage (rIVA/rIVB/rIVC)	2.58 (1.47~4.54)	0.001	3.92 (2.01~7.64)	< 0.001
Early stage (rI/rII/rIII)	1.0 (reference)		1.0 (reference)	
TILs Foxp3+ lymphocytes				
High	1.72 (0.96~3.05)	0.07	1.30 (0.68~2.50)	0.43
Low	1.0 (reference)		1.0 (reference)	
TILs CD8+ lymphocytes				
Low	2.40 (1.37~4.18)	0.002	2.16 (1.14~4.08)	0.02
High	1.0 (reference)		1.0 (reference)	
Recurrent NPC Galectin-9 expression				
High	1.72 (0.96~3.06)	0.07	1.46 (0.78~2.73)	0.23
Low	1.0 (reference)		1.0 (reference)	

Abbreviation: NPC, nasopharyngeal carcinoma; TILs, tumour-infiltrating lymphocytes, HR: hazard ratio.

## Discussion

After we compared immunologic marker expression in the tumour microenvironment between primary and recurrent NPC, we report two interesting findings from our study. The first finding primarily focuses on the immunologic changes between primary and recurrent NPC. In contrast to primary NPC, more than half of the patients with recurrent NPC had increased percentages of Galectin-9+ tumour cells and of Foxp3+ lymphocytes. More than one-third of the patients with recurrent NPC had increased percentages of Tim-3+ and CD4+ lymphocytes. Conversely, nearly half of the patients with recurrent NPC had a decrease in the percentage of CD8+ lymphocytes. Furthermore, the increased percentages of Galectin-9+ tumour cells and Foxp3+ lymphocytes and the decreased percentage of CD8+ lymphocytes significantly differed between primary and recurrent NPC. Finally, the patients with recurrent NPC had a high percentage of Galectin-9+ tumour cells accompanied by a low percentage of CD8+ lymphocytes and high percentages of Foxp3+ and Tim-3+ lymphocytes.

Theoretically, NPC is an EBV-related malignancy. During the process of carcinogenesis, NPC cells still retain functional antigen-presenting machinery. Therefore, the immune escape has been regarded as a specific feature of the tumour microenvironment in NPC^16, 17^. Among several possible immune escape mechanisms, the immune checkpoint Galectin-9/Tim-3 interaction has been reported as one of the most common types in NPC^13, 14, 18, 19^. This immunologic interaction could promote the formation of Foxp3+ lymphocytes and then inhibit the population of CD8+ lymphocytes^20, 21^. Moreover, it has been shown that Galectin-9 was extremely abundant in NPC cells in either xenografted tumours or clinical specimens^19^. Because patients with recurrent NPC in our series had a significantly higher percentage of Galectin-9+ tumour cells, we speculate that the initial treatment may have an important effect on tumour selection. It has been reported that radiation induces dying cells to express significantly more tumour-specific antigens on their surface, resulting in CD8+ lymphocytes being recruited to perform tumour-specific killing^22^. This finding suggests that Galectin-9+ tumour cells in primary NPC may be more resistant to this radiation-induced cytotoxic killing by CD8+ lymphocytes. Subsequently, these Galectin-9+ tumour cells had selective growth advantage in recurrent NPC. In recurrent NPC, to maintain immune escape, these high Galectin-9 expressing NPC cells maintains their ability to inactivate the survival and growth of tumour-infiltrating CD8+ lymphocytes. In addition, this inhibitory effect of CD8+ lymphocytes may be related to the presence of more Foxp3+ lymphocytes in the tumour microenvironment of recurrent NPC^23^.

Our second finding primarily focuses on the impact of immunologic markers in the tumour microenvironment, specifically how a low percentage of CD8+ lymphocytes, high percentage of Galectin-9+ NPC cells and high percentage of Foxp3+ lymphocytes affect the outcome of recurrent NPC. Although a high percentage of Galectin-9+ tumour cells and a high percentage of Foxp3+ lymphocytes in the tumour microenvironment were only marginally significant, a low percentage of CD8+ lymphocytes was a significant risk factor for relapse-free survival and overall survival. Therefore, it is reasonable to speculate that the Galectin-9/Tim-3/Foxp3+ immunologic interaction and its subsequent suppression in CD8+ lymphocytes may play an important role in determining the outcome of salvage treatment. In fact, the association between CD8+ lymphocytes in the tumour microenvironment and treatment outcomes have been reported in NPC and other malignancies^24–27^. Recurrent NPC cells with high Galectin-9 expression had the ability to maintain immune escape by interacting with Tim-3+ lymphocytes^13, 18, 28^. The low percentage of CD8+ lymphocytes in the tumour microenvironment, which is a sign of an immunocompromised status, could serve as an effective biomarker to predict the response to salvage treatment in recurrent NPC.

**Figure 4:**
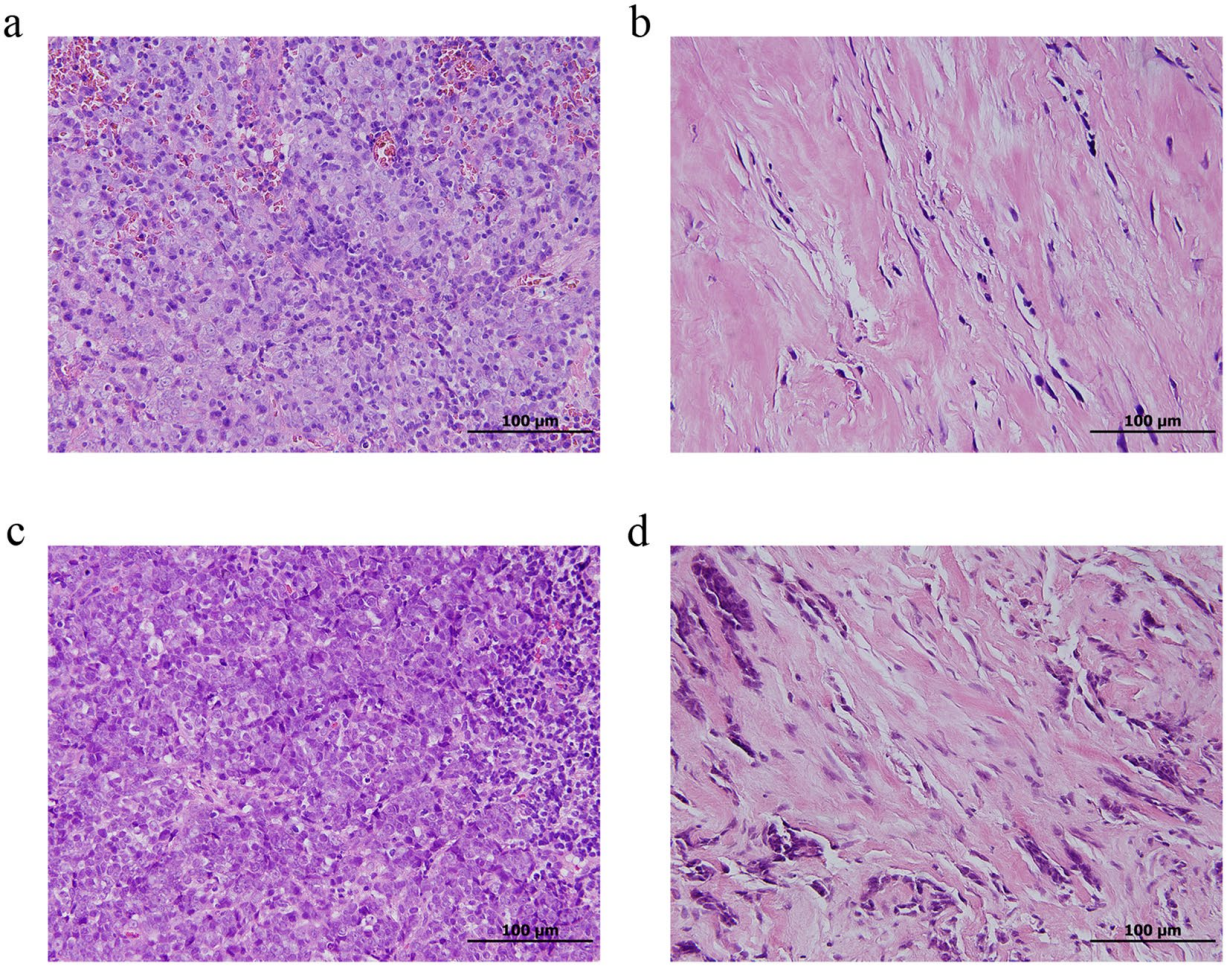
The radiation-associated changes in histopathologic examination. (**a**) Primary nasopharyngeal carcinoma (NPC) without fibrosis. (**b**) Recurrent NPC with marked fibrosis in local nasopharyngeal tissue. (**c**) Primary NPC without fibrosis. (**d**) Recurrent NPC with marked fibrosis in regional neck tissue.

In recurrent NPC, previous radiation could alter the tumour microenvironment and induce tissue fibrosis and microvasculature damage. These changes in physical barriers might play a role in tumour-mediated immune clearance because cells of the adaptive immune system must be able to infiltrate or invade into the tumour to eradicate it. In our series, we frequently identified fibrotic tissue changes (Fig. 4) in the previous radiation field. This radiation-associated physical barrier may partially account for the decreased percentage of CD8+ lymphocytes in recurrent NPC. However, this radiation-associated fibrosis did not prevent the infiltration of Tim-3+ and Foxp3+ lymphocytes in the tumour microenvironment. A recent breakthrough in the treatment of melanoma and other cancers with immune checkpoint inhibitors has rejuvenated enthusiasm in immunotherapy for cancer treatment^29–32^. Currently, the choice of salvage treatment is limited for recurrent NPC. Immunotherapeutic strategies, especially those targeting the Galectin-9/Tim-3/Foxp3 interaction, may serve as the potential salvage treatments for recurrent NPC^33^ in recurrent NPC.

There were some limitations in our study. First, this retrospective study may be vulnerable to selection bias. Although we try to statistically control for this bias using Cox regression model, residual bias may remain. Second, the immunologic characteristics in different histological types, local nasopharynx, regional neck and distant sites, may have originally had different immunologic profiles^34^. These differences may reflect underlying mechanisms in the effect of immunologic changes in recurrent NPC. The strength of this study is that we succeeded in comparing all tissue samples between primary and recurrent NPC in 95 patients. Well-designed prospective case series studies with more patients are necessary in the future to clarify the changes in and the roles of immunologic profiles for recurrent NPC.

## Patients and Methods

### Ethics Statement

This study was approved by the Institutional Review Board of National Taiwan University Hospital. As this was a retrospective analysis of routine data, we requested and were granted a waiver of individual informed consent from the ethics committee. All methods were performed in accordance with the relevant guidelines and regulations. Patient records/information were anonymized and de-identified prior to analyses.

### Patient population

We retrospectively reviewed the medical records of patients who were diagnosed with recurrent NPC (defined as persistent tumour after primary treatment, local/regional neck recurrence or distant metastasis) at the National Taiwan University Hospital between October 2007 and October 2015. The exclusion criteria included patients who had no history of curative treatment for primary NPC in our hospital, patients with primary and/or recurrent NPC diagnosis but without tissue for definite pathologic confirmation, and patients with malignancies other than NPC. Therefore, all the patients in our series had complete primary and recurrent NPC tissue for pathologic comparison. The TNM status of primary and recurrent NPC was classified according to the 2010 criteria of the American Joint Committee on Cancer (AJCC)^35^.

### Immunohistochemical analyses of Galectin-9+ tumour cells and Tim-3+ , CD4+ , CD8+ and Foxp3+ lymphocytes in the tumour microenvironment of primary and recurrent NPC

For immunohistochemical (IHC) staining of Tim-3, Galectin-9, CD4, CD8 and Foxp3, 4-μm-thick sections from each formalin-fixed, paraffin-embedded tissue block were de-waxed with xylene and rehydrated through a graded series of ethanol. Antibodies against Tim-3 (Clone D5D5R, Cell Signalling Technology, Inc., Danvers, MA, USA), Galectin-9 (GeneTex, Inc., Hsinchu City, Taiwan, R.O.C.), CD4 (clone SP35, Spring Bioscience, Inc., Pleasanton, CA, USA), CD8 (clone SP16, Spring Bioscience, Inc., Pleasanton, CA, USA), and Foxp3 (clone 2A11G9, Santa Cruz Biotechnology, Inc., Dallas, Texas, USA) were used for immunohistochemistry. Sections were incubated with anti-Tim-3 antibody (1:300 dilution) for 1 hour; antigen retrieval was performed by autoclaving the sections in Novocastra Epitope Retrieval Solution pH 9.0 (Leica Biosystems Newcastle Ltd, Newcastle Upon Tyne, UK) for 10 min at 121 °C. Sections were incubated with anti-Galectin 9 antibody (1:300 dilution) for 1 hour; antigen retrieval was performed by incubating the sections with Protease 1 (Ventana Medical Systems, Inc., Tucson, Arizona, USA) for 10 min. Sections were incubated with anti-CD4 antibody (1:50 dilution) for 1 hour; antigen retrieval was performed by autoclaving the sections in citrate buffer (pH 6.0) for 10 min at 121 °C. Sections were incubated with anti-CD8 antibody (1:100 dilution) for 1 hour; antigen retrieval was performed by autoclaving the sections in citrate buffer (pH 6.0) for 10 min at 121 °C. Sections were incubated with anti-Foxp3 antibody (1:500 dilution) for 1.5 hour; antigen retrieval was performed by autoclaving the sections in citrate buffer (pH 6.0) for 10 min at 121 °C. The UltraVision Quanto Detection System HRP DAB (Thermo Fisher Scientific, Fremont, CA, USA) was used according to the manufacturer’s instructions. The sections were counterstained with haematoxylin and then mounted. For each stromal infiltrating lymphocyte subgroups, the percentages of lymphocytes compared to the total amount of nucleated cells in the stromal compartment were examined. The scoring of positive lymphocytes and Galectin 9+ tumour cells were estimated by two independent pathologists (Y.-L.C. and C.-T.W.) using a dual-handed microscope by screening the whole immunohistochemical staing sections. High expression of CD4+, CD8+, Tim-3+, Fop3+ lymphocytes and Galectin-9+ tumour cells was defined as values greater than the median percentages.

### Statistical analysis

All immunologic factors were tested for normal distribution by Kolmogorov-Smirnov Test. The Wilcoxon signed-rank test was used to compare the changes in percentages of all immunologic markers between primary and recurrent NPC. The median percentages of all immunologic markers were used as the cut-off points for grouping the patients into high or low level of expression groups. Chi-square tests and Fisher’s exact tests (only in cell number less than five) were used to determine differences in the clinical and immunological characteristics (low or high expression) between the patients with low and high Galectin 9 expression as appropriate. The starting point of the follow-up period was defined as the completion of the curative treatment of primary NPC for each patient. The end point of the follow-up period was either June 2016 or the time when the patient died. The primary outcomes were relapse-free survival and overall survival. All sites of persistent, residual or recurrent tumours after salvage treatment were recorded as failure in terms of the relapse-free survival metric, and all deaths were recorded against the overall survival parameter. The 5-year survival rates of relapse-free and overall survival were calculated by using the Kaplan–Meier product limit method. Significance levels among the curves were determined using the log-rank test. Potential risk factors and pathological characteristics were further analyzed using a multivariate Cox regression model. All statistical analyses were performed using the SPSS software package, version 18.0 (SPSS Inc., Chicago, IL, USA). All statistical tests were two-tailed and corresponding p values < 0.05 were interpreted as statistically significant.
